# High TIL, HLA, and Immune Checkpoint Expression in Conventional High-Grade and Dedifferentiated Chondrosarcoma and Poor Clinical Course of the Disease

**DOI:** 10.3389/fonc.2021.598001

**Published:** 2021-04-12

**Authors:** Sjoerd P. F. T. Nota, Ahmad Al-Sukaini, Shalin S. Patel, Francesco Sabbatino, G. Petur Nielsen, Vikram Deshpande, Jennifer H. Yearley, Soldano Ferrone, Xinhui Wang, Joseph H. Schwab

**Affiliations:** ^1^ Department of Orthopaedic Surgery, Massachusetts General Hospital, Harvard Medical School, Boston, MA, United States; ^2^ Section of Orthopaedic Oncology, Massachusetts General Hospital, Harvard Medical School, Boston, MA, United States; ^3^ Department of Pathology, Massachusetts General Hospital, Harvard Medical School, Boston, MA, United States; ^4^ Department of Translational Medicine, Merck & Co., Inc., Kenilworth, NJ, United States

**Keywords:** PD-L1, B7-H3, HLA class I, T cell infiltration, chondrosarcoma

## Abstract

**Purpose:**

The aim of this study was to characterize chondrosarcoma tumor infiltration by immune cells and the expression of immunologically relevant molecules. This information may contribute to our understanding of the role of immunological events in the pathogenesis of chondrosarcoma and to the rational design of immunotherapeutic strategies.

**Patients and Methods:**

A tissue microarray (TMA) containing 52 conventional and 24 dedifferentiated chondrosarcoma specimens was analyzed by immunohistochemical staining for the expression of parameters associated with tumor antigen-specific immune responses, namely, CD4^+^ and CD8^+^ tumor infiltrating lymphocytes (TILs) and the expression of HLA class I heavy chain, beta-2 microglobulin (β2m), HLA class II and immune checkpoint molecules, B7-H3 and PD-1/PD-L1. The results were correlated with histopathological characteristics and the clinical course of the disease.

**Results:**

CD8^+^ TILs were present in 21% of the conventional and 90% of the dedifferentiated chondrosarcoma tumors tested. B7-H3 was expressed in 69% of the conventional and 96% of the dedifferentiated chondrosarcoma tumors tested. PD-1 and PD-L1 were expressed 53% and 33% respectively of the dedifferentiated tumors tested. PD-L1 expression was associated with shorter time to metastasis.

**Conclusion:**

The tumor infiltration by lymphocytes suggests that chondrosarcoma is immunogenic. Defects in HLA class I antigen and expression of the checkpoint molecules B7-H3 and PD-1/PD-L1 suggest that tumor cells utilize escape mechanisms to avoid immune recognition and destruction. This data implies that chondrosarcoma will benefit from strategies that enhance the immunogenicity of tumor antigens and/or counteract the escape mechanisms.

## Introduction

Conventional chondrosarcoma is the second most common primary malignancy of bone (1). The clinical course of this disease including development of metastases is closely associated with histologic grade. Whereas low-grade conventional chondrosarcoma has a favorable prognosis, high-grade conventional chondrosarcoma, and in particular, dedifferentiated chondrosarcoma, has a poor outcome and a high tendency to metastasize (2, 3). Since conventional radiation and chemotherapy are ineffective, surgical resection is the standard of care for primary conventional and dedifferentiated chondrosarcoma. No effective systemic therapies for the treatment of metastatic disease are available (4, 5).

Major progress has been recently made in the development of immunotherapeutic strategies for the treatment of malignant diseases (6, 7). Impressive clinical responses have been convincingly documented in some of the treated patients with several types of cancer (8, 9). These clinical findings have stimulated interest in the development and application of immunotherapeutic strategies for the treatment of chondrosarcoma (10, 11).

Immunotherapy aims at influencing and/or enhancing a tumor antigen-specific immune response in a host with the expectation that it will eliminate cancer cells. Essentially all elements of the host’s acquired and innate immune system participate in an effective tumor antigen-specific immune response. Preclinical and clinically-based studies have shown beneficial responses to be highly dependent on the induction and functional activity of cytotoxic CD8^+^ T lymphocytes, which recognize tumor antigen(s) expressed by tumor cells. These antigens are processed and presented on tumor cell surface in the context of HLA class I molecules. In turn, CD8^+^ T cells require the functional activities of CD4^+^ T helper cells, which interact with a range of immune cells, such as antigen presenting cells (APC) and macrophages that present tumor antigens in the context of HLA class II molecules (12). However, cancer cells also develop multiple escape mechanisms to avoid immune surveillance and abrogate potentially effective host tumor antigen-specific immune responses. Foremost are defects in antigen processing and presenting machinery, which include defects in HLA class I subunits, namely, HLA class I heavy chain and beta-2 microglobulin (β2m) and in HLA class II molecules (13–15). More recently, attention has been focused on immune checkpoint molecules, which normally help prevent inappropriate targeting of normal cells by the host’s immune system (16). However, many cancer cells have co-opted these checkpoint inhibitors in order to prevent their destruction by CD8^+^ and CD4^+^ tumor infiltrating lymphocytes (TILs) (16). The immune checkpoint molecules, B7-H3 and PD-1/PD-L1, are currently being actively investigated in clinical studies and/or are currently used as targets of therapeutic strategies for the treatment of many human cancer types (17–20). B7-H3 is an immune checkpoint molecule with limited expression in normal tissues (21) which has most commonly been associated with inhibition of cytotoxic CD8^+^ T lymphocyte activation (22) by tumor cells presenting tumor antigens on HLA class I antigen complexes. The other checkpoint molecule, PD-L1, is expressed on normal and cancer cells and appears to release inhibitory signals upon its interaction with its receptor PD-1 on T cells and thereby weakens their reactivity (17, 23).

Limited information is available about the role of immunological events in the pathogenesis and clinical course of chondrosarcoma. The lack of this information has a negative impact on the development of immunotherapies, which have been shown to be very effective in other types of previously recalcitrant cancers. The aim of the current study was to investigate the presence of immune cells and the expression of a selected set of immunologically relevant molecules in a tissue microarray consisting of conventional and dedifferentiated chondrosarcoma specimens and control tissues. The parameters chosen were 1) CD8^+^ and CD4^+^ TILs 2) HLA class I heavy chain and β2m, 3) HLA class II and 4) immune checkpoint B7-H3 and PD-1/PD-L1 molecules. The detection of TILs in the tumor microenvironment would be compatible with the possibility that a host’s immune system recognizes and mounts an immune response against tumor antigens expressed on chondrosarcoma cells. Abnormalities in HLA expression and expression of checkpoint molecules would suggest potential escape mechanisms utilized by chondrosarcoma cells to avoid immune recognition and destruction.

## Patients, Material and Methods

### Patient Characteristics and Tumor Specimens

This study represents a retrospective evaluation of tumor specimens obtained from a cohort of 76 patients with chondrosarcoma with a mean age of 56 years (range, 18-83); 51% were male. They were treated at Massachusetts General Hospital (MGH) during a 20-year period (1993 to 2013). The patients were included in the study if they had a minimum of 2 years of follow-up or until death and sufficient paraffin embedded tumor tissue was available for the construction of the tissue micro-array. Patient information collected on each patient included: age, gender, margin status, tumor grade and presence of metastases. This study was approved by the Institutional Review Board at MGH (approval number 2013P001012). **Table 1** summarizes the clinicopathologic characteristics of the patients and their tumors. The tumors included conventional and dedifferentiated chondrosarcoma as confirmed by 2 senior musculoskeletal pathologists (G.P. N. and V. D.) according to the WHO classification system (24). Of the 76 patients selected for the study, 24 were diagnosed with a dedifferentiated chondrosarcoma.

**Table 1:** Clinical and pathological characteristics of the analyzed chondrosarcoma tumors.

Grade 1. (Sample # = 17)			Grade 2. (Sample # = 30)			Grade 3. (Sample # = 5)			Dedifferentiated (Sample # = 24)		
	**Mean ± SD **	**range**		**Mean ± SD**	**range**		**Mean ± SD**	**range**		**Mean ± SD**	**range**
Age, years (n=17)	49 ± 14	18-69	Age, years (#=30)	57 ± 16	33-83	Age, years (#=5)	56 ± 13	39-73	Age, years (#=24)	59 ± 13	39-82
**Sex**	**#**	**%**	**Sex**	**#**	**%**	**Sex**	**#**	**%**	**Sex**	**#**	**%**
male	6	35	male	15	50	male	3	60	male	15	63
female	11	65	female	15	50	female	2	40	female	9	38
	**Mean ± SD**	**range**		**Mean ± SD**	**range**		**Mean ± SD**	**range**		**Mean ± SD**	**range**
Tumor Size, cm3 (n=14)	137 ± 235	1.2-900	Tumor Size, cm3 (n=25)	705 ± 1253	1.8-5888	Tumor Size, cm3 (n=5)	463 ± 499	36-1248	Tumor Size, cm3 (n=21)	591 ± 875	12-3680
**Anatomic Site**	**#**	**%**	**Anatomic Site**	**#**	**%**	**Anatomic Site**	**#**	**%**	**Anatomic Site**	**#**	**%**
Spine	2	12	Spine	0	0	Spine	1	20	Spine	1	4.2
Scapula/clavicle	3	18	Scapula/clavicle	3	10	Scapula/clavicle	0	0	Scapula/clavicle	2	8.3
Sternum	1	5.9	Sternum	0	0	Sternum	0	0	Sternum	0	0
Rib(s)	0	0	Rib(s)	2	6.7	Rib(s)	0	0	Rib(s)	1	4.2
Humerus	1	5.9	Humerus	5	17	Humerus	0	0	Humerus	2	8.3
Pelvis	1	5.9	Pelvis	5	17	Pelvis	3	60	Pelvis	5	21
Sacrum	2	12	Sacrum	2	6.7	Sacrum	0	0	Sacrum	0	0
Femur	5	29	Femur	10	33	Femur	1	20	Femur	12	50
Tibia	0	0	Tibia	2	6.7	Tibia	0	0	Tibia	1	4.2
Fibula	2	12	Fibula	1	3.3	Fibula	0	0	Fibula	0	0
**Margin**, mm	**#**	**%**	**Margin**, mm	**#**	**%**	**Margin**, mm	**#**	**%**	**Margin**, mm	**#**	**%**
0	3	18	0	4	13	0	2	40	0	8	33
<1	3	18	<1	6	20	<1	2	40	<1	5	21
≥1	8	47	≥1	19	63	≥1	1	20	≥1	10	42
unknown	3	18	unknown	1	3.3	unknown	0	0	unknown	1	4.2
**Extra-osseous extension**	**#**	**%**	**Extra-osseous extension**	**#**	**%**	**Extra-osseous extension**	**#**	**%**	**Extra-osseous extension**	**#**	**%**
yes	9	53	yes	24	80	yes	5	100	yes	22	92
no	6	35	no	6	20	no	0	0	no	1	4.2
unknown	2	12	unknown	0	0	unknown	0	0	unknown	1	4.2
**Metastasis at presentation**	**#**	**%**	**Metastasis at presentation**	**#**	**%**	**Metastasis at presentation**	**#**	**%**	**Metastasis at presentation**	**#**	**%**
yes	0	0	yes	0	0	yes	0	0	yes	7	29
no	17	100	no	30	100	no	5	100	no	17	71
**Metastasis**	**#**	**%**	**Metastasis**	**#**	**%**	**Metastasis**	**#**	**%**	**Metastasis**	**#**	**%**
yes	0	0	yes	6	20	yes	2	40	yes	22	92
no	17	100	no	24	80	no	3	60	no	2	8.3
**Local recurrence**	**#**	**%**	**Local recurrence**	**#**	**%**	**Local recurrence**	**#**	**%**	**Local recurrence**	**#**	**%**
yes	2	12	yes	7	23	yes	1	20	yes	7	29
no	15	88	no	23	77	no	4	80	no	17	71
**Chemotherapy given**	**n**	**%**	**Chemotherapy given**	**n**	**%**	**Chemotherapy given**	**n**	**%**	**Chemotherapy given**	**n**	**%**
yes	0	0	yes	4	13	yes	2	40	yes	16	67
no	17	100	no	26	87	no	3	60	no	8	33
**Radiotherapy**, pre-surgery	**n**	**%**	**Radiotherapy**, pre-surgery	**n**	**%**	**Radiotherapy**, pre-surgery	**n**	**%**	**Radiotherapy**, pre-surgery	**n**	**%**
yes	2	12	yes	6	20	yes	2	40	yes	21	88
no	15	88	no	24	80	no	3	60	no	3	13
**Radiotherapy**, post-surgery	**n**	**%**	**Radiotherapy**, post-surgery	**n**	**%**	**Radiotherapy**, post-surgery	**n**	**%**	**Radiotherapy**, post-surgery	**n**	**%**
yes	5	29	yes	7	23	yes	3	60	yes	10	42
no	12	71	no	23	77	no	2	20	no	14	58

### Chondrosarcoma Tissue Microarray (TMA)

The TMA was constructed using 4-millimeter (mm) diameter cores extracted from representative regions of tumor blocks. In addition to the primary tumor specimens of 76 patients, 8 out of the 76 patients had both primary and corresponding metastatic tissue available for embedding in the TMA. Three of these metastases were from patients with conventional chondrosarcoma and 5 were from patients with dedifferentiated chondrosarcoma. In addition we included 8 randomly selected enchondroma in our TMA. Control tissues of human spleen, human cartilage, human liver, human lymph node, human melanoma metastasis, melanoma xenografts and mouse liver were also included in construction of the TMA. Four mm formalin-fixed, paraffin-embedded tissue sections from the TMA block were used as substrates in immunohistochemical staining. All of the prepared TMA sections contained the full complement of tumor tissue for analysis of each immune parameter being analyzed. The total number of tumor cores suitable for analysis varied due to confined amount of reliable interpretable tissue following specific experiments, as indicated by the number of samples utilized for each marker (in text, figures and tables). When we couldn’t reliably interpret the staining we excluded the data of staining of the particular case from the analysis as indicated.

### Monoclonal Antibodies (mAb)

The human CD8 (clone 4B12) and CD4 (EPR6844) specific mAb were purchased from DAKO (Carpinteria, CA, USA) and Abcam (Cambridge, MA, USA), respectively.

The mAb HC-A2, which recognizes β2m-free HLA-A (excluding -A24), -B7301, and -G heavy chains (25, 26); mAb HC-10, which recognizes β2m-free HLA-A3, -A10, -A28, -A29, -A30, -A31, -A32, -A33, and all β2m-free -HLA-B (excluding -B5702, -B5804, and -B73) and -HLA-C heavy chains (25–27), β2m-specific mAb NAMB1 (28) and mAb LGII-612.14 which recognizes a monomorphic epitope expressed on the β chain of HLA DR, -DQ, and -DP antigens (29), were developed as described before. mAbs were purified from ascitic fluid by affinity chromatography on a Protein G column (GE Healthcare Life Sciences, Pittsburgh, PA). The purity and activity of mAb preparations were controlled by SDS-PAGE and by binding assays with the cognate antigen, respectively. The B7-H3 specific mAb 1027 was purchased from R&D System (Minneapolis, MN, USA) (30–32). The PD-L1-specific mAb clone 22C3 was developed by Merck Research Laboratories; and the PD-1 specific mAb clone NAT105, was purchased from Cell Marque (33).

### Immunohistochemical Staining of Chondrosarcoma TMA

Staining with CD8- and CD4-specific mAb was performed according to the manufacturers’ instructions. Results were calculated by counting the number of stained infiltrating cells within the tumor tissue in a 200x magnification. Lymphocytes were counted in a high-power field that was placed randomly in the tumor tissue. Depending on the amount of tumor tissue the field was placed a maximum of 4 times per tumor core. The total number of lymphocytes was counted and then summed up and was subsequently divided by the number of high-power fields that were counted in the multiple cores in each tumor. This resulted in an actual mean lymphocytic infiltration per high-power-field per tumor.

The immunohistochemical staining of TMA sections with HC-A2, HC-10, NAMB1, LGII-612.14 and B7-H3 mAb was performed as described previously (34). The percentage of stained tumor cells and staining intensity in each lesion were assessed by an investigator who had no knowledge of the patients’ characteristics and clinical outcomes. Results were scored as positive, heterogeneous, or negative when the percentage of stained tumor cells in an entire lesion was greater than 75%, 75% to 25%, and less than 25%, respectively (35). The staining with the anti–PD-1 and anti–PD-L1 mAb was performed by Merck Research Laboratories as described (33). Heat-induced epitope retrieval (HIER) was performed for staining for PD-1 and PD-L1. Slides were immersed in FLEX High pH target retrieval solution for 20 minutes at 97°C (cat. K8012; DAKO). Slides were incubated in 3% hydrogen peroxide solution to block endogenous peroxidase in the tissues before incubating them for 60 minutes at room temperature with the primary antibody. Visualization of the antigen-antibody binding was performed by application of the FLEX+ polymer system (cat. K8012; DAKO) and by application of DAB chromagen (cat. K4368; DAKO). Counterstaining with hematoxylin was used on the stained slides.

Decalcified tissue was excluded from the analysis for PD-1 and PD-L1 since the results of staining decalcified tissue were not reproducible.

### Statistical Analysis

Differences in contingency tables were investigated with Fisher’s exact test and correlations were displayed with Spearman’s rank correlation coefficients. Mann–Whitney U test was applied to investigate differences in two groups of continuous data. To analyze time to survival concerning overall survival and time to metastasis the log-rank test of equality across strata was applied and cox regression analysis was used for continuous variables. The statistical analysis was performed with the use of STATA 12 software, (StataCorp. 2011. Stata Statistical Software: Release 12. College Station, TX: StataCorp LP.).

## Results

### Higher Level of TILs in High-Grade Than in Low-Grade Conventional Chondrosarcoma Tumors

**Figure 1:**
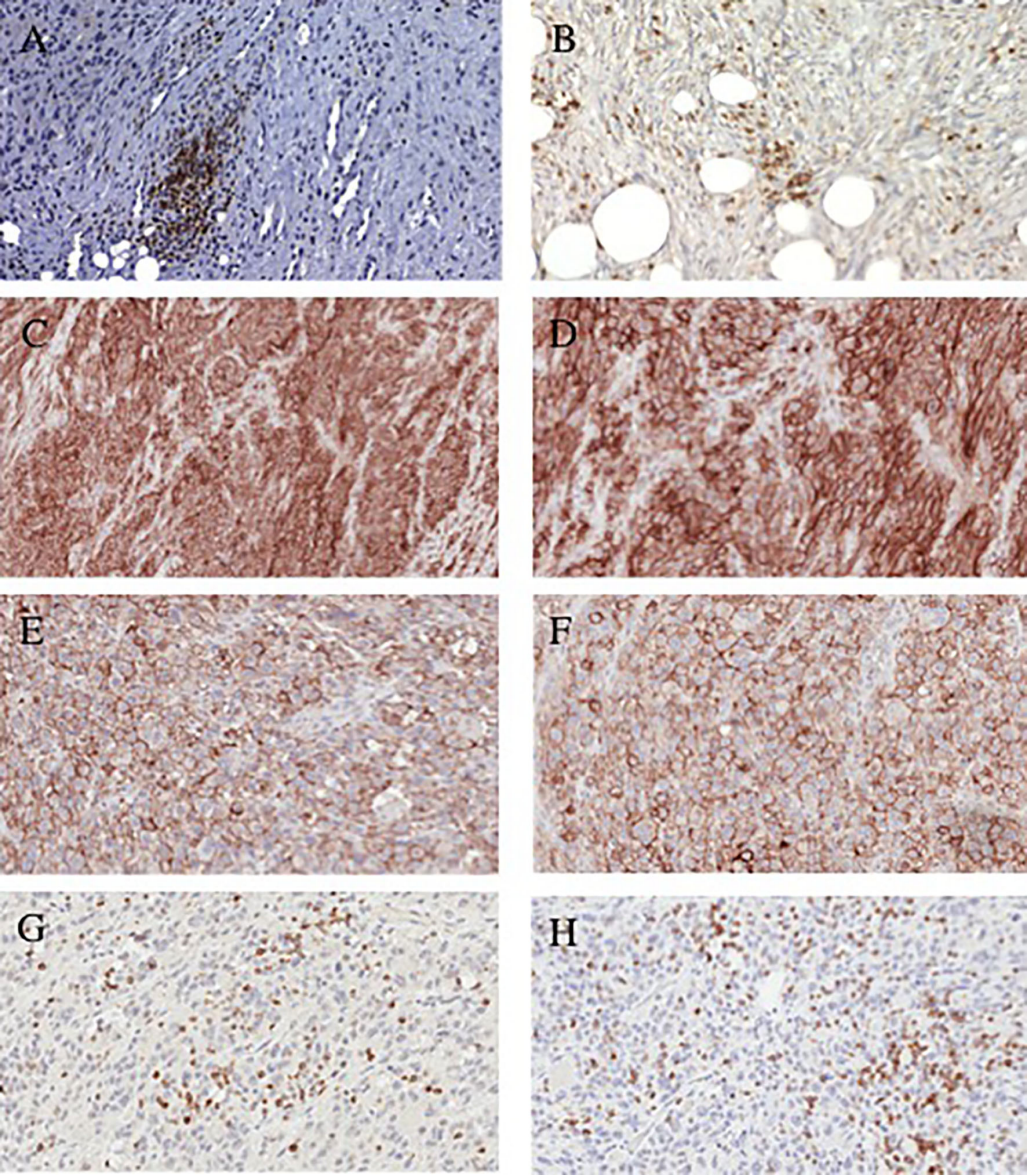
Representative immonohistochemical staining patterns of dedifferentiated chondrosarcoma tumors with lymphocyte and checkpoint-specific monoclonal antibodies. **(A)** CD4^+^ TILs (200x magnification). **(B)** CD8^+^ TILs (200x magnification). **(C)** PD-L1 positive cells (100x magnification). **(D)** PD-L1 positive cells (200x magnification). **(E)** PD-L1 positive cells (200x magnification). **(F)**; PD-L1 positive cells (200x magnification). **(G)** PD-1 positive lymphocytes (200x magnification). **(H)** PD-1 positive lymphocytes (200x magnification).

To investigate whether patients with chondrosarcoma developed an immune response to the tumor antigens expressed by their tumors, the chondrosarcoma TMA was analyzed for the presence of CD4^+^ and CD8^+^ TILs. CD4^+^ T cells were present in 21% of the 61 chondrosarcoma tumors analyzed and CD8^+^ T cells were present in 44% of the 62 chondrosarcoma tumors analyzed (conventional and dedifferentiated combined). The number of CD4^+^ T cells detected ranged between 0.0 and 7.9 lymphocytes per high-power field (mean n=0.16 ± 1.0, n=61), whereas that of CD8^+^ T cells ranged between 0.0 and 52 lymphocytes per high-power field (mean n=5.0 ± 11, n=62) in chondrosarcoma (conventional and dedifferentiated combined). Representative staining patterns of chondrosarcoma tumors with CD4^+^ and CD8^+^ mAb are shown in **Figure 1**. No correlation between levels of CD4^+^ and CD8^+^ TILs was detected (p=0.57). A comparison of the TILs within conventional chondrosarcoma showed that grade 3 tumors had higher CD8^+^ TILs than grade 1 or 2 tumors: mean 3.4 ± 6.1 (range, 0-14, n=5) lymphocytes per high-power field versus 0.26 ± 1.3 (range, 0-7.6, n=37) lymphocytes per high-power field, (p=0.014).

### Higher Level of TILs in Dedifferentiated Chondrosarcoma Than in Conventional Chondrosarcoma Tumors

The level of CD8^+^ TILs in conventional chondrosarcoma tumors was lower than that in the dedifferentiated tumors (21% (9 out of 42 conventional chondrosarcoma analyzed) vs. 90% (18 out of 20 dedifferentiated chondrosarcoma analyzed), P<0.0001). There was no statistical difference in CD4^+^ TILs between conventional and dedifferentiated chondrosarcoma (p=0.90). In a univariable analysis, presence of CD8^+^ TILs in chondrosarcoma (conventional and dedifferentiated combined) was associated with higher risk of mortality (HR=1.1, 95% CI [1.0-1.1], p<0.001). However, in a multivariable analysis controlling for dedifferentiated versus conventional chondrosarcoma, there was no statistical association of CD8^+^ TIL number with survival (HR=1.0, 95% CI [0.98-1.0], p=0.59).

### Higher HLA Class I Expression in High-Grade Than in Low-Grade Conventional Chondrosarcoma Tumors

In conventional chondrosarcoma, HLA-A heavy chain expression was scored positive in 8% (4 out of 50 tumors analyzed), heterogeneous in 42% (21 out of 50 tumors analyzed) and negative in 50% (25 out of 50 tumors analyzed). HLA-B, -C heavy chain expression was scored positive in 23% (12 out of 52 tumors analyzed), heterogeneous in 40% (21 out of 52 tumors analyzed) and negative in 37% (19 out of 52 tumors analyzed) of the tumors (**Table 2**).

**Table 2:** HLA class I subunit and HLA class II antigen expression in benign and malignant cartilage tumors.

		Negative				Heterogeneous				Positive			
**Enchondroma**	HLA	#	Mean (%)	SD (%)	Range (%)	#	Mean (%)	SD (%)	Range (%)	#	Mean (%)	SD (%)	Range (%)
	A Heavy Chain	4	10	0	10-10	4	44	8.7	32-50	0	.	.	.
	B, C Heavy Chain	2	10	0	10-10	3	51	15	37-67	2	75	0	75-75
	B2m	4	10	0	10-10	2	44	1.2	43-45	0	.	.	.
	Class II	6	13	6.1	10-25	0	.	.	.	0	.	.	.
**Grade 1 chondrosarcoma**	HLA	#	Mean (%)	SD (%)	Range (%)	#	Mean (%)	SD (%)	Range (%)	#	Mean (%)	SD (%)	Range (%)
	A Heavy Chain	14	11	2.7	10-20	2	48	18	35-60	0	.	.	.
	B, C Heavy Chain	11	15	5.8	10-25	4	38	9.9	30-50	2	75	0	75-75
	B2m	11	11	2.2	10-17	5	41	9.4	30-52	0	.	.	.
	Class II	15	10	0	10-10	0	.	.	.	0	.	.	.
**Grade 2 chondrosarcoma**	HLA	#	Mean (%)	SD (%)	Range (%)	#	Mean (%)	SD (%)	Range (%)	#	Mean (%)	SD (%)	Range (%)
	A Heavy Chain	10	14	5.6	10-25	15	42	13	27-68	4	79	4.3	75-83
	B, C Heavy Chain	7	13	5.0	10-23	15	46	11	30-63	8	77	4.1	75-85
	B2m	14	16	5.3	10-25	11	46	10	33-67	5	81	5.0	75-87
	Class II	26	13	3.9	10-25	2	41	20	27-55	0	.	.	.
**Grade 3 chondrosarcoma**	HLA	#	Mean (%)	SD (%)	Range (%)	#	Mean (%)	SD (%)	Range (%)	#	Mean (%)	SD (%)	Range (%)
	A Heavy Chain	1	10	.	10-10	4	54	12	36-62	0	.	.	.
	B, C Heavy Chain	1	15	.	15-15	2	68	7.1	63-73	2	78	3.5	75-80
	B2m	1	10	.	10-10	4	45	9.8	35-57	0	.	.	.
	Class II	5	17	5.6	10-25	0	.	.	.	0	.	.	.
**Dedifferentiated chondrosarcoma**	HLA	#	Mean (%)	SD (%)	Range (%)	#	Mean (%)	SD (%)	Range (%)	#	Mean (%)	SD (%)	Range (%)
	A Heavy Chain	0	.	.	.	3	64	9.2	55-73	17	86	5.9	75-90
	B, C Heavy Chain	0	.	.	.	0	.	.	.	20	90	1.5	85-90
	B2m	2	10	0	10-10	10	51	8.8	37-60	7	86	3.0	83-90
	Class II	0	.	.	.	10	52	12	33-70	10	83	6.4	75-90

HLA-A heavy chain expression was significantly higher in grade 2 and grade 3 conventional chondrosarcoma (high-grade) than in grade 1 chondrosarcoma (low-grade): 39% ± 23 (mean expression of 34 grade 2 and grade 3 tumors analyzed, range 10-83%) versus 16% ± 13 (mean expression of 16 grade 1 tumors analyzed, range 10-60%), p=0.0003. Also HLA-B, -C heavy chain expression was significantly higher on average in higher-grade conventional chondrosarcoma than in grade 1 chondrosarcoma: 49% ± 25 (mean expression of 35 grade 2 and grade 3 tumors analyzed, range 10-85%) versus 27% ± 21 (mean expression of 17 grade 1 tumors analyzed, range 10-75%), p=0.0053.

### Higher HLA Class I Expression in Dedifferentiated Than in Conventional Chondrosarcoma Tumors

In dedifferentiated chondrosarcoma, HLA-A heavy chain expression was scored positive in 85% (17 out of 20 tumors analyzed) and heterogeneous in 15% (3 out of 20 tumors analyzed) of the tumors, HLA-B, -C heavy chain expression was scored positive in 100% of the tumors (20 out of 20 tumors analyzed).

**Figure 2:**
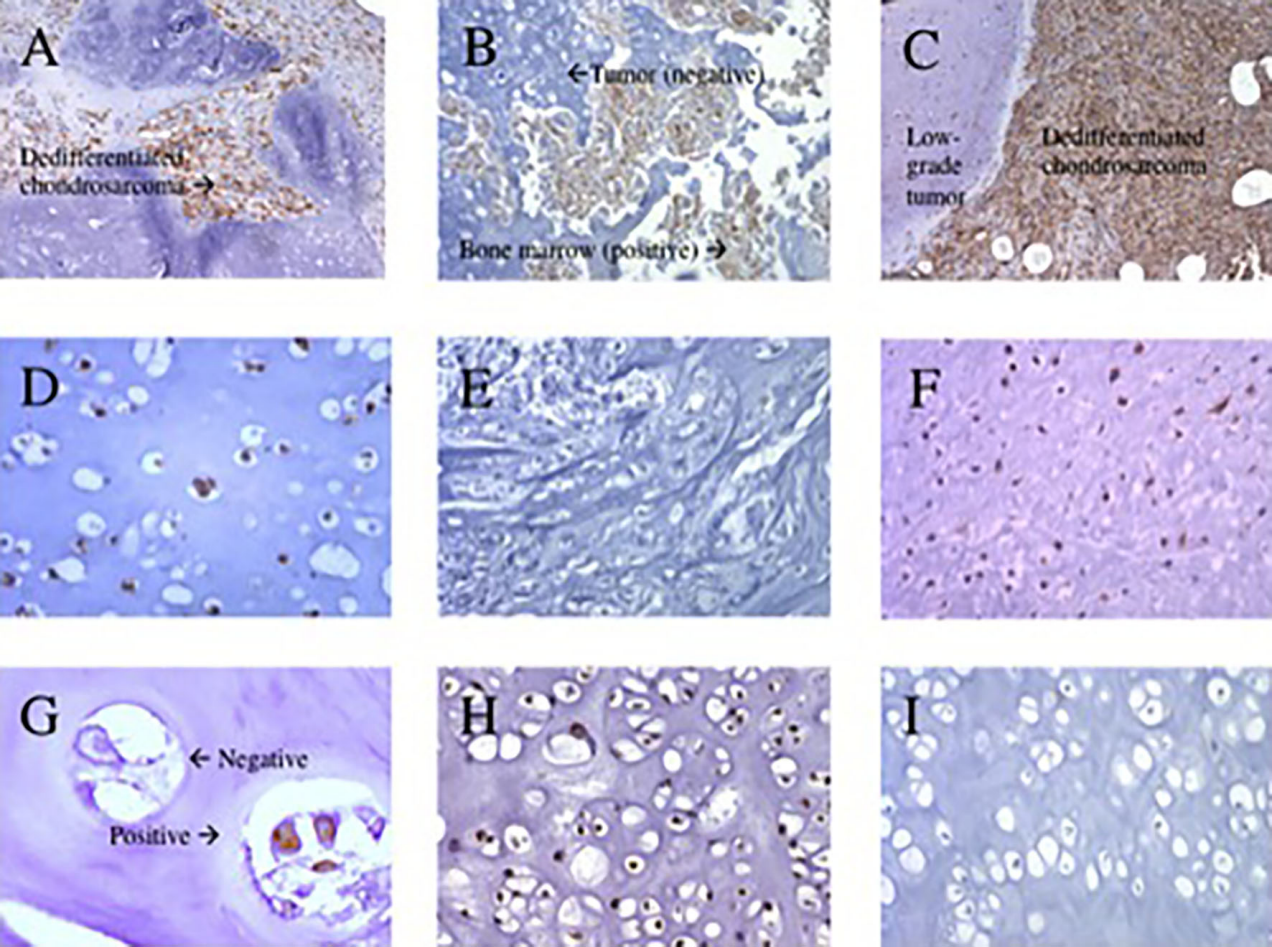
Representative immunohistochemical staining patterns of primary chondrosarcoma tumors with HLA class I subunit specific monoclonal antibodies. **(A)** HLA-A positive stain in dedifferentiated chondrosarcoma (100x magnification). **(B)** HLA-A negative stain in grade 1 chondrosarcoma with positive bone marrow (200x magnification). **(C)** HLA-B, -C positive stain in dedifferentiated chondrosarcoma (100x magnification). **(D)** HLA-B, -C positive stain in grade 2 chondrosarcoma (400x magnification). **(E)** HLA-B, -C negative stain in grade 3 chondrosarcoma (200x magnification). **(F)**; HLA-B, -C positive stain in grade 3 chondrosarcoma (200x magnification). **(G)** β2m both a positive and negative stain in grade 1 chondrosarcoma (400x magnification). **(H)** β2m positive stain in grade 2 chondrosarcoma (200x magnification). **(I)** β2m negative stain in grade 2 chondrosarcoma (200x magnification).

Overall, the mean percentage of HLA-A heavy chain expression was significantly higher in dedifferentiated than in conventional chondrosarcoma: 83% ± 10 (mean expression of the 20 dedifferentiated chondrosarcoma analyzed, range 55-90%) versus 31% ± 23 (mean expression of the 50 conventional chondrosarcoma analyzed, range 10-83%), p<0.0001. Similarly, the mean percentage of HLA-B, -C heavy chain expression was significantly higher in dedifferentiated than in conventional chondrosarcoma: 90% ± 1.5 (mean expression of the 20 dedifferentiated chondrosarcoma analyzed, range 85-90%) versus 42% ± 26 (mean expression of the 52 conventional chondrosarcoma analyzed, range 10-85%), p<0.0001. Representative staining patterns of expression of HLA-A heavy chain with HC-A2 mAb and HLA-B, -C heavy chain with HC-10 mAb are shown in **Figure 2**.

HLA-A heavy chain expression showed a positive and strong correlation with that of HLA-B, -C heavy chain expression (Spearman’s coefficient of 0.88 (n=77), p<0.0001). The presence of CD8^+^ TILs was correlated with HLA-A heavy chain (Spearman’s coefficient of 0.61 (n=68), p<0.0001) and HLA-B, -C heavy chain (Spearman’s coefficient of 0.71 (n=68), p<0.0001).

### Higher β2m Expression in High-Grade Than in Low-Grade Conventional Chondrosarcoma Tumors

In conventional chondrosarcoma β2m expression was scored positive in 10% (5 out of 51 tumors analyzed), heterogeneous in 39% (20 out of 51 tumors analyzed) and negative in 51% (26 out of 51 tumors analyzed) of the tumors analyzed. In addition, the mean percentage of β2m expression was significantly lower in grade 1 tumors than in grade 2 and grade 3 chondrosarcoma: 21% ± 15 (mean expression of 16 grade 1 tumors analyzed, range 10-52%) versus 38% ± 24 (mean expression of 35 grade 2 and grade 3 tumors analyzed, range 10-87%), p=0.0048. Representative staining patterns with β2m-specific NAMB1 mAb are shown in **Figure 2**.

### Higher β2m Expression in Dedifferentiated Chondrosarcoma Than in Conventional Chondrosarcoma Tumors

In dedifferentiated chondrosarcoma β2m expression was scored positive in 37% (7 out of 19 tumors analyzed), heterogeneous in 53% (10 out of 19 tumors analyzed) and negative in 11% (2 out of 19 tumors analyzed) of the tumors (**Table 2**). This pattern was similar to that found for HLA class I heavy chains in these 2 types of chondrosarcoma. The mean percentage of β2m expression was significantly lower in conventional chondrosarcoma than in dedifferentiated tumors: 32% ± 23 (mean expression of the 51 conventional chondrosarcoma analyzed, range 10-87%) versus 59% ± 25 (mean expression of the 19 dedifferentiated chondrosarcoma, range 10-90%), p=0.0002. Analysis of conventional chondrosarcoma showed that β2m expression is positively and significantly correlated with that of HLA-A heavy chain (Spearman’s coefficient of 0.72, p<0.0001) and of HLA-B, -C heavy chain (Spearman’s coefficient of 0.79, p<0.0001).

### Higher HLA Class II Expression in High-Grade Than in Low-Grade Conventional Chondrosarcoma Tumors

In conventional chondrosarcoma, HLA class II expression was scored heterogeneous in 4% (2 out of 48 tumors analyzed) and negative in 96% (46 out of 48 tumors analyzed). HLA class II expression was lower in grade 1 tumors than in grade 2 and 3 chondrosarcoma: 10% ± 0 (mean expression of 15 grade 1 tumors analyzed, range 10-10%) versus 15% ± 8.7 (mean expression of 33 grade 2 and grade 3 tumors analyzed, range 10-55%), p=0.0005.

### Higher HLA Class II Expression in Dedifferentiated Chondrosarcoma Than in Conventional Chondrosarcoma Tumors

In differentiated chondrosarcoma, HLA class II expression was scored positive in 50% (10 out of 20 tumors analyzed) and heterogeneous in 50% (10 out of 20 tumors analyzed) (**Table 2**).

Overall, HLA class II expression was significantly higher in dedifferentiated chondrosarcoma than in conventional chondrosarcoma: 67% ± 19 (mean expression of 20 dedifferentiated chondrosarcoma analyzed, range 33-90%) versus 14% ± 7.5 (mean expression of 48 conventional chondrosarcoma analyzed, range 10-55%), p<0.0001.

### Higher HLA Class I, β2m and HLA Class II Expression in the Dedifferentiated Component Than in the Conventional Component Within Dedifferentiated Chondrosarcoma

In dedifferentiated chondrosarcoma, the HLA-A heavy chain expression of the conventional (low-grade) component was scored positive in 13% (2 out of 15 tumors analyzed), heterogeneous in 20% (3 out of 15 tumor analyzed) en negative in 67% (10 out of 15 tumors analyzed).

The HLA-B, -C heavy chain expression of the conventional component was scored positive in 17% (2 out of 12 tumors analyzed), heterogeneous in 17% (2 out of 12 tumors analyzed) and negative in 67% (8 out of 12 tumors analyzed).

The β2m expression of the conventional component within the dedifferentiated chondrosarcoma was scored positive in 7.7% (1 out of 13 tumors analyzed), heterogeneous in 23% (3 out of 13 tumors analyzed) and negative in 69% (9 out of 13 tumors analyzed).

The HLA class II expression of the conventional component was scored negative in 100% (13 out of 13 tumors analyzed).

Matched analysis of the expression of HLA class I, β2m and HLA class II between the conventional component versus the dedifferentiated component showed higher HLA-A (p=0.0030), HLA-BC (p=0.014), β2m (p=0.050) and HLA class II (p=0.0076) in the dedifferentiated component versus the conventional component in the same tumor.

### Higher B7-H3 Expression in Dedifferentiated Chondrosarcoma Than in Conventional Chondrosarcoma Tumors

In conventional chondrosarcoma, B7-H3 expression was scored positive in 38% (18 out of 48 tumors analysed), heterogeneous in 31% (15 out of 48 tumors analysed) and negative in 31% (15 out of 48 tumors analysed). In dedifferentiated chondrosarcoma, B7-H3 expression was scored positive in 88% (21 out of 24 tumors analysed), heterogeneous in 8% (2 out of 24 tumors analysed) and negative in 4% (1 out of 24 tumors analysed), (**Table 3**). In conventional chondrosarcoma, there were significant differences among 3 B7-H3 expression levels (negative, heterogeneous and positive) and tumor grade (p<0.0001). Higher-grade tumors showed higher B7-H3 expression levels.

**Table 3A:** Higher B7-H3 expression in dedifferentiated than in conventional chondrosarcoma.

	Sample #	Negative Staining	Heterogeneous Staining	Positive Staining
Grade 1	15	11	3	1
Grade 2	28	3	11	14
Grade 3	5	1	1	3
Dedifferentiated	24	1	2	21

### Higher PD-L1 in Dedifferentiated Chondrosarcoma Tumors Than in Conventional Chondrosarcoma Tumors

Eight out of the 15 (53%) patients analyzed with dedifferentiated tumors expressed the immune checkpoint PD-1 antigen on their TILs (excluding the 9 patients with decalcified tissue) (**Table 3**). The mean expression of these 8 patients was 2.8 ± 1.5 (range, 1-5) on a scale to 5. PD-L1 was expressed in 5 out of the 15 dedifferentiated tumors (33%) with a mean expression of 4.8 ± 0.45 (range, 4-5) on a scale to 5 (**Table 3**). PD-1 expression was correlated with that of PD-L1 expression in the dedifferentiated chondrosarcoma (Spearman’s rho = 0.80, p<0.0003) (**Table 3**).

Representative staining patterns of PD-1 and PD-L1 are shown in **Figure 2**. No staining by the PD-L1-specific mAb was detected in any of the analyzed conventional chondrosarcoma (n=29, excluding decalcified tissue). We did not stain the conventional chondrosarcoma for PD-1 since none of the conventional chondrosarcoma expressed any PD-L1 (**Table 3**).

Positive staining of PD-L1 was associated with a significantly shorter time to metastasis (p=0.019) (excluding the patients with metastasized disease at the time of presentation). The mean time to metastasis was 1.6 months, 95% CI [0.032-3.1], for the tumors stained by PD-L1 specific mAb tumors (n=2) but 20 months, 95% CI [1.8-95] for the tumors with no detectable PD-L1 staining (n=12).

### Comparison of the Immunohistochemical Staining Profile of Primary and Autologous Metastatic Chondrosarcoma Lesions in Conventional and Dedifferentiated Chondrosarcoma

The expression of HLA class I antigens, HLA class II antigens and checkpoint molecules in 8 primary and autologous metastatic lesions was compared. Of the 8 primary lesions, 5 were dedifferentiated, 2 were grade 2 and 1 was grade 3 conventional chondrosarcoma. HLA class I and HLA class II expression in the autologous metastases appeared to be higher than in the primary tumors; however, this difference was not statistically significant and was not associated with higher TILs in metastases. No difference in mean PD-1 and PD-L1 expression was detected between primary and autologous metastatic lesions.

Due to the low number of cases, a meaningful statistical analysis was not possible, but a descriptive table of the results of the analysis of the 8 cases stratified according to tumor grade is part of **Table 4**.

**Table 4:** TIL, HLA Class I subunit and HLA Class II antigen expression in metastasis and in primary tumors.

A. Combined dedifferentiated and conventional chondrosarcoma combined										
	**Primary**				**Metastasis**				
	**#**	**Mean (cell #)**	**SD (%)**	**Range (%)**	**#**	**Mean (cell #)**	**SD (%)**	**Range (%)**	**Observation**	**P-value**
CD4^+^	61	0.16	1.0	0-7.9	7	0.080	0.11	0-0.25	Lower	0.090
CD8^+^	62	5.0	11	0-52	6	2.7	4.4	0-11	Lower	0.16
**HLA**	**#**	**Mean (%)**	**SD (%)**	**Range (%)**	**#**	**Mean (%)**	**SD (%)**	**Range (%)**		
A Heavy Chain	70	46	31	10-90	6	66	27	20-90	Higher	0.59
B, C Heavy Chain	72	55	31	10-90	7	83	7.6	75-90	Higher	0.17
B2m	70	40	26	10-90	7	62	24	30-90	Higher	0.22
Class II	68	29	27	10-90	7	53	33	10-83	Higher	0.17
**B. Conventional chondrosarcoma**										
** Primary**					**Metastasis**					
	**#**	**Mean (cell #)**	**SD (%)**	**Range (%)**	**#**	**Mean (cell #)**	**SD (%)**	**Range (%)**	**Observation**	**P-value**
CD4^+^	39	0.030	0.059	0-0.20	3	0.10	0.091	0-0.17	Higher	0.17
CD8^+^	42	0.63	2.5	0-14	3	0	NA	0-0	Lower	NA
**HLA**	**#**	**Mean (%)**	**SD (%)**	**Range (%)**	**#**	**Mean (%)**	**SD (%)**	**Range (%)**		
A Heavy Chain	50	31	23	10-83	3	49	30	20-80	Higher	0.29
B, C Heavy Chain	52	42	26	10-85	3	78	5.8	75-85	Higher	0.11
B2m	51	32	23	10-87	3	57	25	30-80	Higher	0.29
Class II	48	14	7.5	10-55	3	19	16	10-38	Higher	0.78
**C. Dedifferentiated chondrosarcoma**										
**Primary**					**Metastasis**					
	**#**	**Mean (cell #)**	**SD (%)**	**Range (%)**	**#**	**Mean (cell #)**	**SD (%)**	**Range (%)**	**Observation**	**P-value**
CD4^+^	22	0.39	1.7	0-7.9	4	0.063	0.13	0-0.25	Lower	0.32
CD8^+^	20	14	15	0-52	3	5.4	5.1	0.5-11	Lower	0.18
**HLA**	**#**	**Mean (%)**	**SD (%)**	**Range (%)**	**#**	**Mean (%)**	**SD (%)**	**Range (%)**		
A Heavy Chain	20	83	10	55-90	3	83	7.6	75-90	Equal	0.65
B, C Heavy Chain	20	90	1.5	85-90	4	86	7.5	75-90	Lower	0.32
B2m	19	59	25	10-90	4	67	26	30-90	Higher	0.65
Class II	20	67	19	33-90	4	78	4.1	75-83	Higher	0.18

## Discussion

Our study tested whether patients with chondrosarcoma develop a T cell immune response against their own tumor and we have analysed the expression of immunologically relevant molecules on chondrosarcoma cells. The latter include HLA class I subunits, HLA class II antigens and checkpoints B7-H3 and PD-L1. The resulting information contributes to our understanding of the role of immune surveillance. We showed that low-grade (grade 1) conventional chondrosarcoma are less immunogenic as indicated by limited TILs and lower HLA class I and HLA class II expression compared to high-grade conventional chondrosarcoma (grade 2 and 3). In addition, dedifferentiated chondrosarcoma are more immunogenic compared to conventional chondrosarcoma as indicated by more TILs and higher HLA class I and HLA class II expression.

In this study, HLA class I antigen expression was decreased in 77-92% of the conventional chondrosarcoma, but in only 0-15% of the dedifferentiated chondrosarcoma. As observed in most, if not all the other types of solid cancer analysed (13, 36) defects in HLA class I antigen expression have been found in chondrosarcoma. In chondrosarcoma, the frequency of defects is significantly lower in subtypes with an aggressive phenotype and poor clinical course than in those with a benign phenotype and with a more favourable clinical course. This pattern is at variance with what has been found in most other types of solid cancer. In the latter defective HLA class I expression is in general associated with poor clinical course of the disease (13).

CD8^+^ TILs were present in 90% of dedifferentiated chondrosarcoma and 21% of conventional chondrosarcoma with high-grade conventional tumors having more CD8^+^ TILs than low-grade chondrosarcoma. It is intriguing that dedifferentiated chondrosarcoma carry an isocitrate dehydrogenase-1 (IDH1) and isocitrate dehydrogenase-2 (IDH2) mutation (37) with higher frequency than conventional chondrosarcoma since in glioma the neoantigens derived from IDH-mutation have been shown to induce a cellular immune response (38–42). Its inability to control tumor growth in chondrosarcoma may reflect the negative impact of the suppressive tumor microenvironment, especially since in colorectal cancer an association has been found between B7-H3 and IDH1 expression level (43).

Several mechanisms can be envisioned for the association between defective HLA class I antigen expression and favourable course of the disease we have unexpectedly found in chondrosarcoma. One possibility is that immunosurveillance does not play a role in the pathogenesis and clinical course of the disease in chondrosarcoma. However this possibility is unlikely, since the lymphocyte infiltration we have found in tumors indicates that hosts recognize and develop a T cell mediated immune response to tumor antigens expressed by their own tumors. An alternative possibility is that in chondrosarcoma NK cells and not T cells play the major role in the elimination of malignant cells. If so, this mechanism would not be unique of chondrosarcoma, since it has already been described in other cancers (44, 45).

In this case high HLA class I antigen expression would represent a defensive mechanism for malignant cells, since it would very effectively inhibit the ability of NK cells to eliminate malignant cells. One last possibility that we favour is that patients mount an immune response to the tumor antigens expressed by their own tumors, however this response has no antitumor activity because it is inhibited by an immunosuppressive microenvironment. Our study demonstrated that the immune checkpoint molecule B7-H3 is expressed in 96% of dedifferentiated tumours and in 69% of conventional chondrosarcoma. Interestingly, B7-H3 expression was associated with high-grade versus lower grade conventional chondrosarcoma similar to our findings with HLA class I antigen expression. B7-H3 has been shown to inhibit the antitumor activity of T cells in many types of solid cancer has been associated with immune suppression and worse prognosis in multiple cancers, including glioblastoma, lung, renal cell carcinoma, and pancreatic ductal adenocarcinoma cancer (31, 46–48). B7-H3 has been shown to exert an inhibitory immune effect by preventing activation of CD8^+^ T lymphocytes (49). With a preferential expression on tumor cells and limited expression on normal tissue, B7-H3 is an attractive target for cancer immunotherapy (21, 50).

Another interesting result of our study is the association of PD-L1 expression on chondrosarcoma cells in primary tumors with shorter time to metastatic spreading. Caution has to be exercised in interpreting this finding because the number of tumor samples analysed is small, however in other cancers PD-L1 expression has been associated with metastatic disease (51–53).

Therefore, this association needs to be independently confirmed by analysing a large number of additional samples. If this association is corroborated by additional results and reflects a cause effect relationship, then one potential mechanism for the metastatic spreading is the escape of tumor cells from immunosurveillance because of T cell exhaustion caused by the inhibitory signals triggered by the interactions between PD-1 expressed on T cells and PD-L1 expressed on chondrosarcoma cells and macrophages in the tumor microenvironment.

In our study, PD-L1 was expressed by 33% (5/15) of the dedifferentiated chondrosarcoma. In conventional chondrosarcoma there was no PD-L1 expression independently of tumor grade. Our findings are consistent with the information in the literature (54)

One might ask whether the results we have described have any clinical relevance especially in the area of therapy since this is an unmet need in chondrosarcoma. Our results can contribute to the rational design of immunotherapeutic strategies for the treatment of chondrosarcoma, especially since immunosurveillance appears to play a role in its clinical course. Therapies targeting B7-H3 and PD-1/PD-L1 axis may have a beneficial effect on this malignancy, since they may counteract the immunosuppression induced by B7-H3 and the PD-1 axis and may inhibit the metastatic potential of chondrosarcoma cells. This possibility is supported by two recent studies which have reported clinical responses in two patients with chondrosarcoma following treatment with nivolumab (55) or pembrolizumab (56).

## Data Availability Statement

The raw data supporting the conclusions of this article will be made available by the authors, without undue reservation.

## Ethics Statement

The studies involving human participants were reviewed and approved by Partners Human Research Committee Institutionals Review Board: Protocol #: 2013P001012. Written informed consent for participation was not required for this study in accordance with the national legislation and the institutional requirements.

## Author Contributions

All authors have made substantial contributions to all of the following: (1) the conception and design of the study, or acquisition of data, or analysis and interpretation of data, (2) drafting the article or revising it critically for important intellectual content, (3) final approval of the version to be submitted. All authors contributed to the article and approved the submitted version.

## Conflict of Interest

JY reports other from Merck, outside the submitted work; In addition, JY has a patent Immunohistochemical proximity assay for PD-1 positive cells and PD-Ligand positive cells in tumor tissue pending, and a patent Antibodies that bind to human programmed death ligand 2 (PD-L2) pending. JY was employed by Merck & Co., Inc.

The remaining authors declare that the research was conducted in the absence of any commercial or financial relationships that could be construed as a potential conflict of interest.
